# Effect of Different Nuclear Localization Signals on the Subcellular Localization and Anti-HIV-1 Function of the MxB Protein

**DOI:** 10.3389/fmicb.2021.675201

**Published:** 2021-05-20

**Authors:** Keli Chai, Zhen Wang, Qinghua Pan, Juan Tan, Wentao Qiao, Chen Liang

**Affiliations:** ^1^Key Laboratory of Molecular Microbiology and Technology, Ministry of Education, College of Life Sciences, Nankai University, Tianjin, China; ^2^Lady Davis Institute, Jewish General Hospital, Montreal, QC, Canada; ^3^Department of Microbiology and Immunology, McGill University, Montreal, QC, Canada; ^4^Department of Medicine, McGill University, Montreal, QC, Canada

**Keywords:** MxB, HIV-1, nuclear localization signal, antiviral activity, interferon

## Abstract

Interferon exerts its antiviral activity by stimulating the expression of antiviral proteins. These interferon stimulate genes (ISGs) often target a group of viruses with unique molecular mechanisms. One such ISG is myxovirus resistance B (MxB) that has been reported to inhibit human immunodeficiency virus type 1 (HIV-1) by targeting viral capsid and impairing nuclear import of viral DNA. The antiviral specificity of MxB is determined by its N-terminal 25 amino acids sequence which has the nuclear localization activity, therefore functions as a nuclear localization signal (NLS). In this study, we report that the bipartite NLS, but not the classic NLS, the PY-NLS, nor the arginine-rich NLS, when used to replace the N-terminal sequence of MxB, drastically suppress HIV-1 gene expression and virus production, thus creates a new anti-HIV-1 mechanism. MxB preserves its anti-HIV-1 activity when its N-terminal sequence is replaced by the arginine-rich NLS. Interestingly, the arginine-rich NLS allows MxB to inhibit HIV-1 CA mutants that are otherwise resistant to wild type MxB, which suggests sequence specific targeting of viral capsid. Together, these data implicate that it is not the nuclear import function itself, but rather the sequence and the mechanism of action of the NLS which define the antiviral property of MxB.

## Introduction

Interferons are produced in response to viral infections, and provide one main mechanism of host innate antiviral defense (Bonjardim, 2005; Randall and Goodbourn, 2008). Interferons operate by inducing the expression of hundreds of genes, collectively called interferon-stimulated genes (ISGs), many of which have direct antiviral activities. The myxovirus resistance (Mx) genes are typical ISGs, and were discovered for their protection of mice from lethal infection by influenza virus (Staeheli et al., 1986; Pavlovic et al., 1992).

Humans have two *Mx* genes, *MxA* and *MxB* (also called *MX1* and *MX2*), both of which have strong and distinct antiviral properties (Haller and Kochs, 2011; Haller et al., 2015). The MxA protein is mainly localized within the cytoplasm, inhibits a wide range of viruses, including influenza A virus, vesicular stomatitis virus, LaCrosse virus, hepatitis B virus and others (Pavlovic et al., 1990; Kochs et al., 2002; Haller and Kochs, 2011; Li et al., 2012). Initial studies did not reveal significant antiviral activity of MxB. In 2013, three groups reported that MxB inhibits HIV-1 by impairing nuclear entry of viral DNA (Goujon et al., 2013; Kane et al., 2013; Liu et al., 2013). MxB was subsequently shown to inhibit herpesvirus (Crameri et al., 2018; Jaguva Vasudevan et al., 2018; Schilling et al., 2018), hepatitis B virus (Wang et al., 2020), and hepatitis C virus (Yi et al., 2019).

Both MxA and MxB are large GTPase, share similar structures composed of a globular GTPase domain which is linked to a stalk domain by the BSE (bundle signaling element) hinge (Gao et al., 2011; Fribourgh et al., 2014; Haller et al., 2015; Alvarez et al., 2017). The distinct antiviral specificities of MxA and MxB are the result of the different strategies they use to recognize viral targets (Gao et al., 2011; Kong et al., 2014; Patzina et al., 2014; Schulte et al., 2015; Verhelst et al., 2015). MxA uses the loop 4 motif in the stalk domain to target the nucleocapsid protein of influenza A virus (Mitchell et al., 2012; Patzina et al., 2014; Verhelst et al., 2015), whereas MxB depends on its N-terminal domain (NTD) to intercept HIV-1 capsid (Goujon et al., 2014; Kong et al., 2014; Goujon et al., 2015; Schulte et al., 2015; Smaga et al., 2019). For example, attaching the 90-amino acid NTD of MxB to the N-terminus of MxA confers thus modified MxA the ability of inhibiting HIV-1 infection (Goujon et al., 2014).

In addition to its interaction with HIV-1 capsid core in concert with the GTPase and the stalk domains, the NTD of MxB also has the nuclear localization function (Matreyek et al., 2014; Goujon et al., 2015; Kane et al., 2018). This NTD, when attached to the heterologous proteins, can direct their localization to the nucleus (Kane et al., 2018). However, MxB is mainly seen on the nuclear envelope and within the cytoplasm, which likely results from the action of a potential nuclear export signal (NES) in MxB, or strong interaction of MxB with nuclear pore complex (NPC) components (King et al., 2004; Busnadiego et al., 2014; Goujon et al., 2014; Dicks et al., 2018; Kane et al., 2018; Xu et al., 2020). In any event, the nuclear envelope location does not appear to be crucial for MxB inhibition of HIV-1 infection, since certain MxB mutants, such as the MxBΔ1-25 + NLS mutant and MX2(N91)-ARFAPTIN 2 mutant, lost localization to the nuclear envelope, yet still exhibited strong anti-HIV-1 activity (Matreyek et al., 2014; Kane et al., 2018). It is likely that localization to the nuclear envelope may be required for certain cellular functions of MxB.

The NTD NLS of MxB does not fit in the definition of classic NLS, the bipartite NLS, nor the PY-NLS. Nonetheless, its nuclear localization function does depend on importin-β, but exhibits clear difference in the requirement of nucleoporins as compared to the classic NLS of SV40 large T antigen (Kane et al., 2018). The pressures for human MxB to evolve and maintain such an NLS are unknown. It is worth noting that the MxB proteins of primates all keep this NLS in their NTDs (Busnadiego et al., 2014). To further understand this conservation of NLS in MxB proteins, we changed the MxB NLS to other well-characterized NLS sequences and examine which heterologous NLS is able to at least partially rescue the known functions of MxB including inhibition of HIV-1. We found that the bipartite NLS is able to restore MxB localization to the nuclear envelope, whereas the arginine-rich NLS enables MxB to inhibit HIV-1, albeit exhibiting different restriction profiles toward HIV-1 capsid mutants.

## Materials and Methods

### Plasmid DNA

DNA sequence encoding wild type human MxB (GenBank accession number NM_002463.1) was cloned into either pEGFP-N1 vector (Clontech) with green fluorescence protein (GFP) sequence attached to the C-terminus or pQCXIP retroviral vector (Clontech) with a Flag-tag at the C-terminus. The MxBΔ(1-25) mutation was generated by deleting sequences from Met1 to Glu25. The different NLS-MxBΔ(1-25) chimeras were generated by replacing the first 25 amino acids with corresponding heterogeneous NLS sequences, the different N1-NLS constructs were generated by inserting the corresponding NLS sequences into pEGFP-N1 vector, and primers used are listed in Table 1. The HIV-1 proviral DNA clone NL4-3 was obtained from the NIH AIDS Reagent Program. HIV-1 DNA mutants NL4-3/CA-P90T, NL4-3/CA-E187V, and NL4-3/CA-P207S were generated using site-directed mutagenesis PCR method (Clontech). Tat-HA was cloned into pcDNA3.1 expression vector (Invitrogen) with a C-terminal HA tag. Luciferase reporter constructs CMV-Luc, SV40-Luc, PFV-LTR-Luc, and HIV-LTR-Luc were generated by inserting the promoter sequences into pGL3-basic luciferase reporter vector (Promega), which lacks promoter and enhancer sequences, expression of luciferase activity depends on insertion of a functional promoter at the upstream of luciferase gene. The importin-β siRNA (ID s7918) was purchased from Ambion. All constructs were confirmed by sequencing.

**TABLE 1:** Primers for generating MxB mutants.

Primer	Sequence (5′–3′)
SV40T-NLS-MxBΔ(1–25)-F	CTAGCGATGCCAAAAAAGAAGAGAAAGGTAGGGCCAAAAAAGAAGAGAAAGGTAGGGC
SV40T-NLS-MxBΔ(1–25)-R	TCGAGCCCTACCTTTCTCTTCTTTTTTGGCCCTACCTTTCTCTTCTTTTTTGGCATCG
BI-NP-NLS-MxBΔ(1–25)-F	CTAGCGATGAAGAGACCTGCAGCCACCAAAAAGGCAGGCCAGGCAAAGAAGAAGAAAC
BI-NP-NLS-MxBΔ(1–25)-R	TCGAGTTTCTTCTTCTTTGCCTGGCCTGCCTTTTTGGTGGCTGCAGGTCTCTTCATCG
BI-RB-NLS-MxBΔ(1–25)-F	CTAGCGATGAAAAGAAGTGCTGAAGGAAGCAACCCTCCTAAACCACTGAAAAAACTACGCC
BI-RB-NLS-MxBΔ(1–25)-R	TCGAGGCGTAGTTTTTTCAGTGGTTTAGGAGGGTTGCTTCCTTCAGCACTTCTTTTCATCG
BI-RAC3-NLS-MxBΔ(1–25)-F	CTAGCGATGCGAAAACGCAAATTGCCATGTGATACTCCAGGACAAGGTCTTACCTGCAGTGGTGAAAAACGGAGACGGC
BI-RAC3-NLS-MxBΔ(1–25)-R	TCGAGCCGTCTCCGTTTTTCACCACTGCAGGTAAGACCTTGTCCTGGAGTATCACATGGCAATTTGCGTTTTCGCATCG
M9-NLS-MxBΔ(1–25)-F	CTAGCAATGGGGAATTACAACAATCAGTCTTCAAATTTTGGACCCATGAAGGGAGGAAATTTTGGAGGCAGAAGCTCTGGCCCCT ATGGCGGTGGAGGCCAATACC
M9-NLS-MxBΔ(1–25)-R	TCGAGGTATTGGCCTCCACCGCCATAGGGGCCAGAGCTTCTGCCTCCAAAATTTCCTCCCTTCATGGGTCCAAAATTTGAA GACTGATTGTTGTAATTCCCCATTG
Tat-NLS-MxBΔ(1–25)-F	CTAGCAATGGGCAGGAAGAAGCGGAGACAGCGACGAAGAGCTCCTCAGGACAGTCAGACTCATCAAC
Tat-NLS-MxBΔ(1–25)-R	TCGAGTTGATGAGTCTGACTGTCCTGAGGAGCTCTTCGTCGCTGTCTCCGCTTCTTCCTGCCCATTG
Rev-NLS-MxBΔ(1–25)-F	CTAGCGATGACCCGACAGGCCCGAAGGAATAGAAGAAGAAGGTGGAGAGAGAGACAGAGAC
Rev-NLS-MxBΔ(1–25)-R	TCGAGTCTCTGTCTCTCTCTCCACCTTCTTCTTCTATTCCTTCGGGCCTGTCGGGTCATCG

*F, Forward primer; R, Reverse primer.*

### Cell Culture and Transfection

HEK293T, HeLa, and TZM-bl cells were cultured in Dulbecco’s Modified Eagle Medium (DMEM) (Invitrogen) supplemented with 10% fetal bovine serum (FBS) (Invitrogen), 50 U/ml penicillin, and 50 μg/ml streptomycin (Invitrogen). SupT1 cells were grown in RPMI 160 medium (Invitrogen) supplemented with 10% FBS, 2 mM L-glutamine, 50 U/ml penicillin, and 50 μg/ml streptomycin. Transfection of plasmid DNAs or siRNAs was conducted with polyethyleneimine (PEI) (Sigma-Aldrich) or Lipofectamine 3000 (Invitrogen) according with the manufacturer’s instructions.

### Immunofluorescence and Confocal Microscopy

HeLa cells were seeded onto 20 mm diameter glass coverslips and transfected. The transfected cells were fixed with 4% paraformaldehyde (in PBS) for 15 min at room temperature, washed with PBS, and permeabilized with 0.5%Triton-X 100 in PBS for 10 min, then incubated in blocking buffer (5% BSA and 5% FBS in PBS) for 1 h. Cells were sequentially stained with mouse anti-importin-β antibody (Invitrogen, catalog number MA3-070, 1:500 dilution) or rabbit anti-FLAG antibody (Cell Signaling Technology, catalog number 14793, 1:1,000 dilution) for 2 h, and with Alexa Fluor 647 conjugated goat anti-mouse secondary antibody (Invitrogen, catalog number A21237, 1:500 dilution) or Alexa Fluor 594 conjugated goat anti-rabbit secondary antibody (Invitrogen, catalog number A11037, 1:500 dilution) for 1 h. Nuclei was stained with 0.5 μg/ml DAPI (4′,6-diamidino-2-phenylindole) (Thermo Fisher Scientific) for 20 min. Slides were covered with the mounting medium (Thermo Fisher Scientific). Images were recorded with Leica confocal microscope using a 63x oil objective.

### Western Blotting

The transfected cells were harvested and lysed in RIPA buffer containing protease inhibitor cocktail (Roche) for 30 min on ice. The proteins in the cell lysates were separated on 10% polyacrylamide gel by SDS–polyacrylamide gel electrophoresis (SDS-PAGE), and transferred onto polyvinylidene difluoride (PVDF) membranes (Roche). The membranes were blocked with 5% skim milk in PBS, followed by incubation with primary antibodies. The primary antibodies used include rabbit anti-HIV-1 p24 antibody (Sigma Aldrich, catalog number SAB3500946, 1:5,000 dilution), mouse anti-GFP antibody (Invitrogen, catalog number MA5-15256, 1:5,000 dilution), mouse anti-FLAG antibody (Sigma Aldrich, catalog number F1804-200UG, 1:5,000 dilution), mouse anti-HIV-1 Nef antibody (NIH AIDS Reagents program, catalog number 1539, 1:5,000 dilution), mouse anti-GAPDH antibody (Santa Cruz Biotechnology, catalog number sc-32233, 1:5,000 dilution), and mouse anti-Tubulin antibody (Santa Cruz Biotechnology, catalog number sc-23948, 1:5,000 dilution). The membranes were further incubated with horseradish peroxidase (HRP) conjugated-Goat anti-mouse (SeraCare, catalog number 5450-0011, 1:10,000 dilution) or anti-rabbit (SeraCare, catalog number 5450-0010, 1:10,000 dilution) secondary antibodies. Bands were detected by using chemiluminescence reagent Plus-ECL Western blotting substrate (PerkinElmer, catalog number NEL105001EA) and exposure to X-ray films (Carestream, catalog number 6041768).

### Luciferase Reporter Assay

Transfected or infected cells were collected and lysed in cell culture lysis buffer (Promega). Luciferase activity was measured using the luciferase assay system (Promega). All experiments were performed in triplicate and repeated at least three times.

### Viral Reverse Transcriptase Assay

10 μl viral supernatants were mixed with 40 μl freshly prepared reaction mixture including 50 mM Tris-HCl (pH 7.9), 150 mM KCl, 5 mM MgCl_2_, 0.5 mM EGTA, 0.05% Triton X-100, 5 mM DTT, 0.3 mM GSH, 0.025 U poly(rA)/oligo(dT) (Midland Certified Reagent Company, catalog number P-4012), and 5 U Ci [^3^H] TTP (PerkinElmer, catalog number NET221A005 MC). After 3 h incubation at 37°C, the newly synthesized DNA was precipitated by adding 150 μl 10% TCA at 4°C for 30 min. Then the [^3^H]-labeled DNA was collected by passing the reaction mixture through multiscreen glass fiber FC plate (Millipore), and measured using the Beckman Scintillation Counter. The level of [^3^H]-labeled DNA indicates the amount of newly synthesized DNA by viral reverse transcriptase.

### Virus and Infection

HIV-1 particles were produced by transfection of HEK293T cells with proviral DNA constructs NL4-3, NL4-3/CA-P90T, NL4-3/CA-E187V, or NL4-3/CA-P207S. The culture medium was changed at 6 h post-transfection. Viruses in the supernatants were harvested at 48 h post-transfection, and clarified by centrifugation at 3,000 rpm for 10 min at 4°C. The amount of virus was determined by measuring HIV-1 reverse transcriptase activity. For infection assays, equal amounts of viruses were used to infect SupT1 cell lines for 4 h at 37°C in presence of 5 μg/ml polybrene, after which virus inoculate were washed off. At 48 h post-infection, the number of infected SupT1 cells was determined by flow cytometry after staining with FITC-conjugated anti-p24 antibody. The amounts of infectious HIV-1 viruses in the culture supernatants were determined by infecting the TZM-bl indicator cells and measuring luciferase activity. All infection experiments were performed in triplicate, and repeated at least three times.

### RT-qPCR to Quantify HIV-1 RNA

Analysis of HIV-1 RNA by RT-qPCR was performed as follows. Briefly, total cellular RNA was extracted using Trizol (Invitrogen), followed by DNase (Invitrogen) treatment to remove DNA. Equal amounts of DNase-treated RNA were subjected to reverse transcription for cDNA synthesis using random hexamers (Invitrogen, catalog 8080127) and the MuMLV reverse transcriptase (Invitrogen, catalog number 28025013). Reverse transcription products were quantified by qPCR using Fast SYBR Green Master mix (Invitrogen, catalog 4385612) according to the manufacturer’s instructions. qPCR reactions were performed in triplicate. β-actin mRNA also quantified in each sample to normalize HIV-1 RNA levels. The sequences of primers are as follows: 5′-GAC GCT CTC GCA CCC ATC TC-3′ and 5′-CTG AAG CGC GCA CGG CAA-3′ to quantify HIV-1 full-length RNA, and 5′-GAG CGG TTC CGC TGC CCT GAG GCA CTC-3′ and 5′-GGG CAG TGA TCT CCT TCT GCA TCC TG-3′ to quantify β-actin mRNA.

### Statistical Analysis

Statistical difference between the two groups was analyzed using the Student’s *t*-test with GraphPad Prism version 8.0. Differences were considered statistically significant when the *p*-value was <0.05. In the figures, *p*-values are indicated as follows: ^∗^ for *p* < 0.05, ^∗∗^ for *p* < 0.001, ^∗∗∗^ for *p* < 0.0001, ns for not significant.

## Results

### The Bipartite NLS Supports MxB Localization to the Nuclear Envelope

**FIGURE 1:**
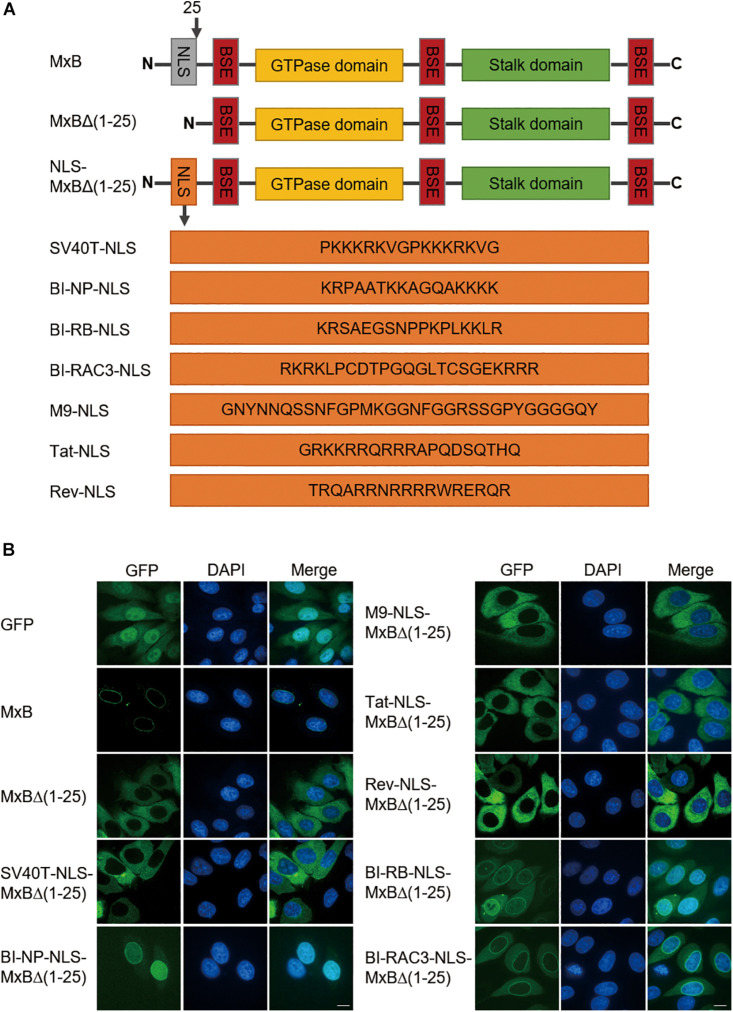
The bipartite NLS supports MxB localization to the nuclear envelope. **(A)** Schematic representation of wild type full-length human MxB, N-terminal truncated MxBΔ(1–25), and MxB variants with N-terminal 25 amino acids replaced by heterologous NLS. The amino acid sequences of NLS are shown. NLS, nuclear localization signal. BSE, bundle signaling element. **(B)** HeLa cells were transfected with either wild type MxB or MxB mutants bearing a C-terminal GFP tag. The subcellular localization of MxB and its mutants was visualized by confocal laser scanning microscopy. Scale bars represent 10 μm.

We first asked which type of NLS, when used to replace the first 25 amino acids sequence of MxB, can support MxB localization to the nuclear envelope. We started with the well-characterized classic NLS of SV40 large T antigen (SV40T-NLS), the bipartite NLS of nucleoplasmin (NP) (BI-NP-NLS), the PY-NLS of hnRNPA1 (M9-NLS) (Lange et al., 2007). We also tested the arginine-rich non-classic NLS sequences of HIV-1 Tat and Rev proteins (Tat-NLS and Rev-NLS), given the presence of the arginine cluster in the NTD of MxB (Figure 1A). The green fluorescence protein (GFP) sequence was attached to the C-terminus of MxB to facilitate detection by confocal microscopy. Similar to what was shown for HA-tagged MxB (Kane et al., 2013), MxB-GFP was mainly seen at the nuclear envelope (Figure 1B). Deletion of the N-terminal 25 amino acids led to diffused cytoplasmic distribution (Figure 1B). None of the SV40T-NLS, M9-NLS, Tat-NLS, or Rev-NLS, when used to replace the 25-amino acid NTD of MxB, restored localization of MxB to the nuclear envelope. In contrast, the BI-NP-NLS rendered complete nuclear localization of the engineered MxB mutant (Figure 1B). To test whether this observation is specific to NP-NLS or is a general feature of bipartite NLS, we further examined the bipartite NLS sequences of retinoblastoma (RB) protein and Ras-related C3 botulinum toxin substrate 3 (RAC3) protein (Cherezova et al., 2011). The results showed that both BI-RB-NLS and BI-RAC3-NLS enabled MxB localization to the nuclear envelope concurrent with dispersed distribution within the cytoplasm (Figure 1B). Therefore, the bipartite NLS, not the other NLS sequences tested herein, is able to position MxB to the nuclear envelope, with notable differences in the overall subcellular localization of the engineered MxB variants between the bipartite NLS sequences tested.

### Nuclear Localization of BI-NP-NLS-MxB Depends on Importin-β

**FIGURE 2:**
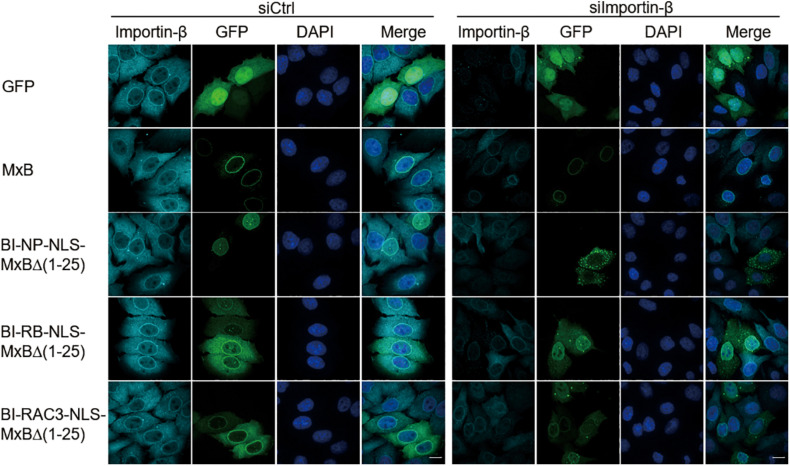
Nuclear localization of BI-NP-NLS-MxBΔ(1–25) depends on importin-β. MxB or MxB mutants bearing a C-terminal GFP tag were expressed in HeLa cells that were transfected with control siRNA (siCtrl) or siRNA targeting importin-β (siImportin-β). Endogenous importin-β was immuno-stained with anti-importin-β antibodies. The subcellular localization of MxB and importin-β was visualized by confocal laser scanning microscopy. Scale bars represent 10 μm.

The bipartite NLS functions by binding the importin-α/β complex (Pawlowski et al., 2010). The nuclear localization activity of MxB NTD, when attached to reporter protein GFP-LacZ, is also dependent on importin-β (Kane et al., 2018). We thus knocked down importin-β with siRNA and examined how the subcellular localization of MxB and its mutants BI-NP-NLS-MxBΔ(1−25), BI-RB-NLS-MxBΔ(1−25), and BI-RAC3-NLS-MxBΔ(1−25) were affected. In agreement with the study by Kane et al. (2018), depletion of importin-β did not affect localization of MxB to the nuclear envelope (Figure 2). Among the three MxB mutants tested, the BI-NP-NLS-MxBΔ(1−25) mutant changed its location from the nucleus to the cytoplasm and formed aggregates, whereas the BI-RB-NLS-MxBΔ(1–25) and BI-RAC3-NLS-MxBΔ(1–25) mutants still showed strong association with nuclear envelope, and moderately increased nuclear presence in importin-β knockdown cells (Figure 2). Therefore, BI-NP-NLS-mediated localization to the nucleus is sensitive to importin-β knockdown, as opposed to the NTD sequence of MxB, BI-RB-NLS, or BI-RAC3-NLS whose activity in directing MxB localization to the nuclear envelope is not affected.

### The Bipartite NLS-Bearing MxB Dramatically Suppresses HIV-1 Gene Expression

**FIGURE 3:**
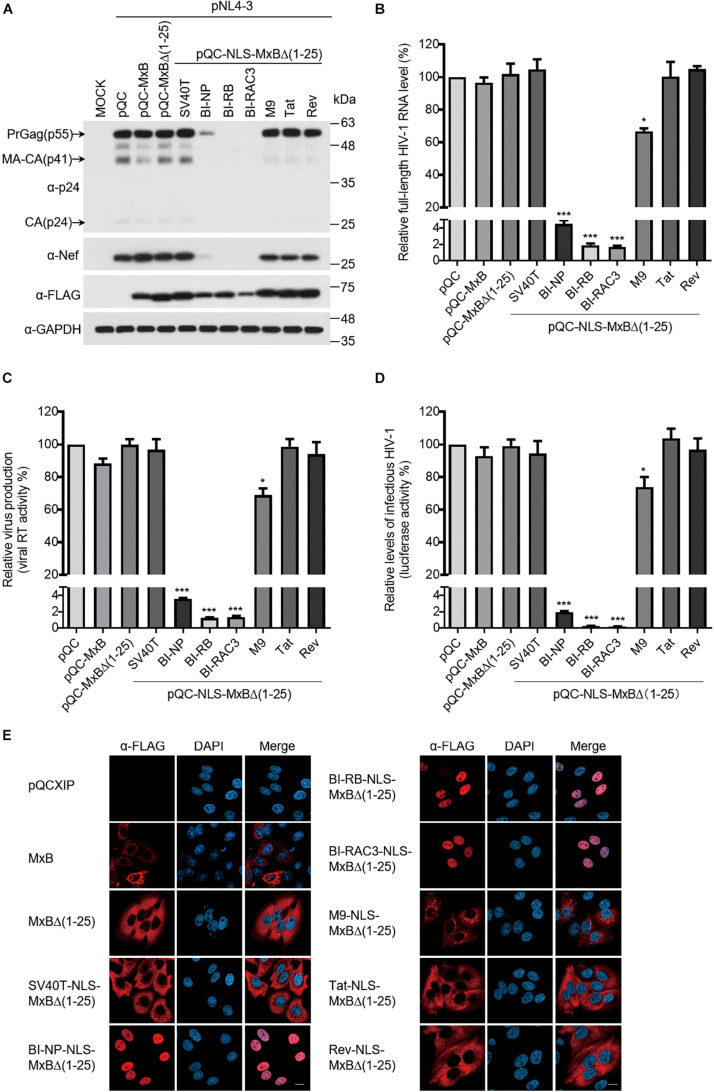
The bipartite NLS-bearing MxB dramatically suppresses HIV-1 gene expression. **(A)** HEK293T cells were transfected with wild type or mutant MxB DNA bearing a C-terminal Flag tag together with HIV-1 NL4-3 proviral DNA. At 48 h post-transfection, lysates of transfected cells were subjected to Western blotting to examine the levels of Gag/p24, Nef, and MxB. GAPDH was also examined as the internal control. **(B)** Total RNA was extracted from the above transfected cells. The levels of full-length HIV-1 RNA were determined by RT-qPCR, and the data were normalized to the levels of β-actin mRNA. Data shown are the average from three independent transfection experiments. **(C)** The levels of viral RT activity in the culture supernatants were determined, the results report the amounts of HIV-1 particles that were produced by the transfected HEK293T cells. Data shown are the average of three independent experiments. **(D)** The levels of infectious HIV-1 in the culture supernatants were determined by infecting TZM-bl indicator cells using the same volume of supernatants. Data shown are averages from three independent experiments. **(E)** HeLa cells were transfected with either wild type pQCXIP-MxB or its mutants bearing a C-terminal FLAG tag. The subcellular localization of MxB and its mutants was visualized by immunofluorescence staining and confocal microscopy. Representative images are shown. Scale bars represent 10 μm.

MxB itself does not affect viral gene expression and virus production when both MxB and HIV-1 DNA are transfected into HEK293T cells (Liu et al., 2013). We posited that changing the NTD to different NLS sequences might not endow MxB with the ability of modulating HIV-1 gene expression. Indeed, the levels of HIV-1 Gag expression were not affected by MxB and its variants containing the NLS of SV40T, M9, Tat or Rev in HEK293T cells that were transfected with HIV-1 DNA and the above MxB DNA clones (Figure 3A), nor the levels of HIV-1 RNA and the amounts of HIV-1 particles were affected (Figures 3B–D). Unexpectedly, all three bipartite NLS transformed MxB into a strong suppressor of HIV-1 Gag expression (Figure 3A). This inhibition occurred at the viral RNA level, since a dramatic reduction in the full-length HIV-1 RNA, was detected with RT-PCR (Figure 3B). As a result, BI-NLS-bearing MxB led to more than 100-fold decrease in the production of HIV-1 particles (Figures 3C,D). We further examined the subcellular localization of MxB-Flag and its mutants by immunofluorescence staining, and observed nuclear localization of BI-NP-NLS, BI-RB-NLS, and BI-RAC3-NLS-bearing MxB (Figure 3E).

**FIGURE 4:**
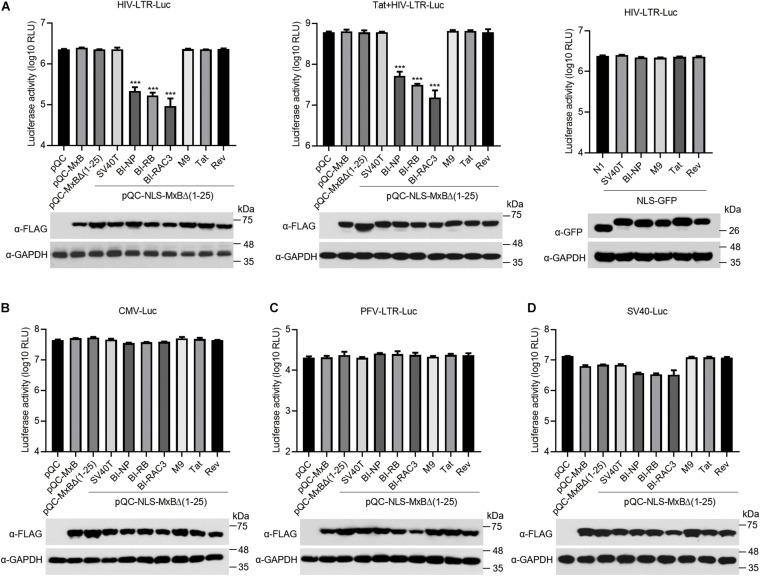
The bipartite NLS-bearing MxB suppresses HIV-1 LTR promoter activity. **(A)** HEK293T cells were co-transfected with HIV-LTR-Luc DNA containing a luciferase reporter gene driven by the HIV-LTR promoter, together with MxB or its mutant DNA (left panel). Same transfections were performed by including HIV-1 Tat DNA (middle panel). HIV-LTR-Luc DNA was also co-transfected with GFP DNA bearing different types of NLS (right panel). At 48 h post-transfection, the transfected cells were collected to measure luciferase activity which is presented in log10 RLU (relative light unit). Expression of MxB or NLS-GFP was examined by Western blotting. Data shown are the average from three independent experiments. **(B–D)** HEK293T cells were co-transfected with MxB DNA, CMV-Luc DNA **(B)**, or SV40-Luc DNA **(C)**, or PFV-LTR-Luc DNA **(D)**. Each of these reporter DNA construct contains a luciferase reporter gene driven by the CMV, SV40 or PFV-LTR promoter. At 48 h post-transfection, the cells were lysed to measure luciferase activity. Expression of MxB and its mutants in the transfected cells was detected by Western blotting. Data shown are the average from three independent experiments.

HIV-1 RNA synthesis is initiated from the viral LTR promoter, and is dramatically enhanced by viral Tat protein. We thus examined the effect of MxB and its mutants on the transcription from HIV-1 LTR and the transactivation activity of viral Tat, by transfecting HEK293T cells with the HIV-1 LTR-Luc reporter DNA, MxB or its mutants, with or without Tat DNA. The results showed that the BI-NLS-bearing MxB inhibited luciferase expression from HIV-1 LTR by more than 10-fold, whereas MxB and other mutants did not exhibit any effect (Figure 4A, left panel). The Tat protein increased luciferase expression from HIV-1 LTR by 250-fold. The magnitude of stimulation by Tat was not affected by MxB, nor its mutants, including bipartite NLS-bearing MxB mutants (Figure 4A, middle panel). But the overall gene expression from HIV-1 LTR in the presence of Tat was reduced by 10-fold by BI-NLS-MxB proteins. To demonstrate that BI-NLS needs to act together with the MxB sequence to inhibit HIV-1 LTR promoter, we attached BI-NLS to GFP and measured the effect of NLS-GFP fusion proteins on HIV-1 LTR transcription. None of the NLS-GFP proteins affected the activity of HIV-1 LTR (Figure 4A, right panel).

We next asked whether the inhibitory effect of BI-NLS-MxB is specific for HIV-1 LTR promoter. We thus transfected the HEK293T cells with MxB DNA and luciferase reporter DNA carrying promoters from cytomegalovirus (CMV), prototype foamy virus (PFV) or SV40. The results showed that neither MxB nor its BI-NLS-MxB mutants markedly changed the levels of luciferase expression from CMV promoter or PFV LTR promoter (Figures 4B,C). In contrast, the level of luciferase expression from SV40 promoter decreased to 50% by MxB, MxB Δ(1–25) or SV40T-NLS-MxBΔ(1–25), to 25% by the BI-NLS-MxBΔ(1–25) mutants. The M9-NLS-MxBΔ(1–25), Tat-NLS-MxBΔ(1–25) and Rev-NLS-MxBΔ(1–25) did not show significant effects (Figure 4D). These data suggest that the BI-NLS-MxBΔ(1–25) proteins specifically inhibit viral gene expression from HIV-1 LTR promoter, thus suppress viral protein expression and virus production.

### The Search for MxB Orthologs Carrying Bipartite NLS

**FIGURE 5:**
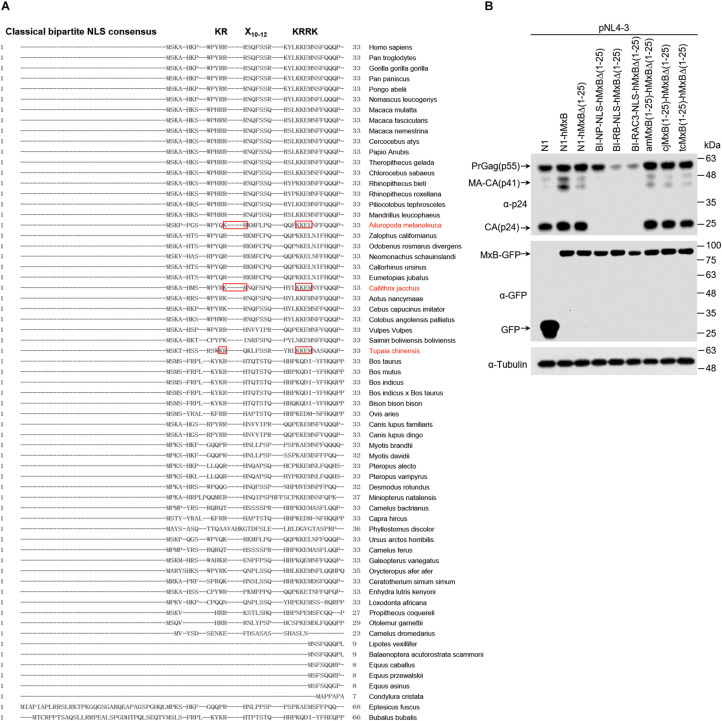
The search for MxB orthologs carrying bipartite NLS. **(A)** Alignment of N-terminal sequences of MxB proteins from 65 species. The bipartite NLS consensus sequence is depicted in bold at the top of the aligned sequences. Highlighted in red boxes are the N-terminal sequences of MxB proteins from three species that contains bipartite-like NLS. **(B)** HEK293T cells were transfected with wild type or mutant MxB DNA together with HIV-1 NL4-3 proviral DNA. At 48 h post-transfection, the transfected cells were subjected to Western blotting to examine the levels of Gag/p24 and MxB. Tubulin was detected as the internal control.

The drastic inhibition of HIV-1 gene expression by the MxB mutants carrying bipartite NLS prompted us to search for MxB orthologs that may carry bipartite NLS at their N-terminal regions. We aligned the N-terminal sequences of MxB proteins from 65 species, which are available in the NCBI database, and found bipartite-like NLS in MxB proteins from three species, Ailuropoda melanoleuca, Callithrix jacchus, and Tupaia chinensis (Figure 5A). We then changed the first 25 amino acids sequence of human MxB to each of the corresponding MxB sequences from these three species. When these MxB mutants were co-transfected into HEK293T cells with HIV-1 DNA, no effect on HIV-1 Gag/p24 expression was detected by Western blotting, whereas the biparpite NLS-bearing MxB mutants drastically decreased HIV-1 Gag/p24 levels (Figure 5B). Thus, unlike the bipartite NLS, the N-terminal sequences of MxB from species Ailuropoda melanoleuca, Callithrix acchus, and Tupaia chinensis do not transform human MxB into a strong inhibitor of HIV-1 gene expression.

### MxB Mutants Bearing the Arginine-Rich NLS Inhibit HIV-1 Infection, but Exhibit Different Sensitivity to Viral CA Mutations

**FIGURE 6:**
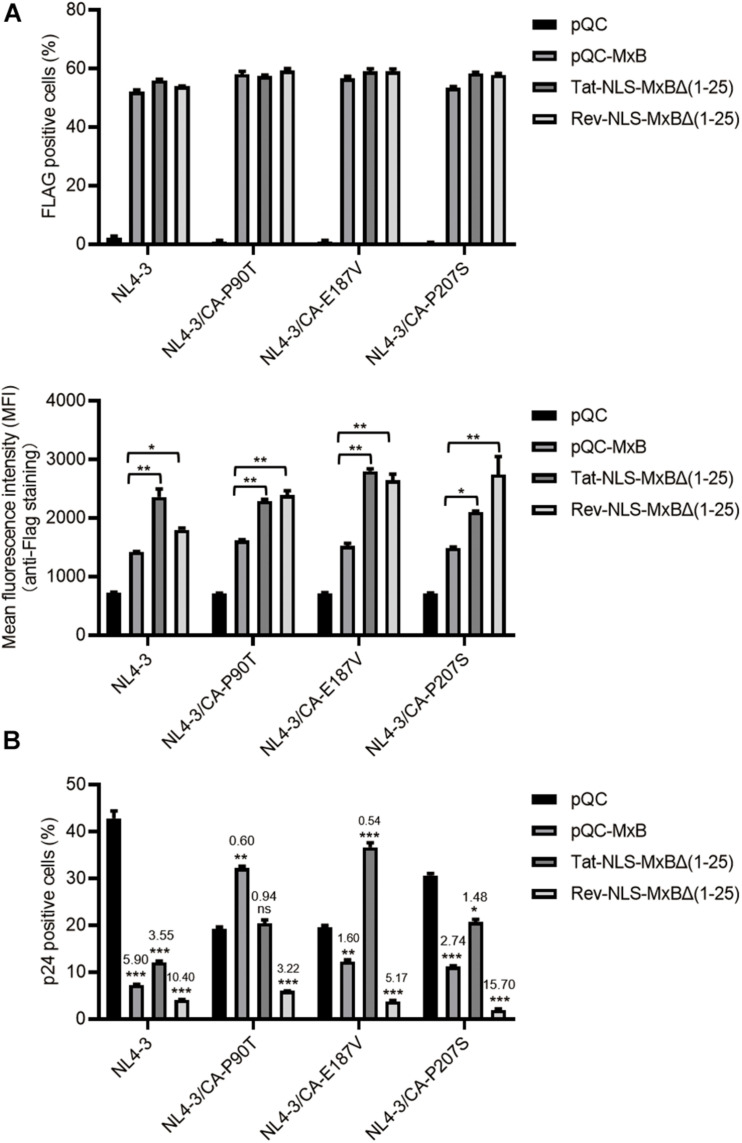
MxB and its mutants bearing the arginine-rich NLS differentially inhibit wild type HIV-1 and viral mutants having CA mutations. SupT1 cells were transduced with retroviral particles that express MxB, Tat-NLS-MxBΔ(1–25), or Rev-NLS-MxBΔ(1–25), followed by infection with HIV-1 and its mutants CA-P90T, CA-E187V, or CA-P207S. MxB and its mutants contain a FLAG tag at the C-terminus. **(A)** Expression of MxB and its mutants was detected by anti-FLAG immunostaining. The percentages of FLAG-positive cells were scored by flow cytometry, and mean fluorescence intensity (MFI) values of FLAG staining were calculated and presented in the bar graphs. Results shown are the average of three independent experiments. *p < 0.05, **p < 0.001, ***p < 0.0001, ns, not significant. **(B)** HIV-1 infected cells were detected by immunostaining viral p24. The percentages of viral p24 positive cells in the Flag-positive cell population were scored by flow cytometry. Results shown are the average of three independent experiments. Fold of inhibition of HIV-1 infection by MxB or its mutants were calculated with reference to the infection of control cells by wild type HIV-1 or viral mutants. *p < 0.05, **p < 0.001, ***p < 0.0001. ns, not significant.

We next tested whether these MxB mutants inhibit HIV-1 infection of CD4^+^ T cells. We used the retroviral vectors to transduce CD4^+^ SupT1 cells, in order to express MxB and its mutants. The results showed that the M9-NLS-MxBΔ(1–25) and the BI-NLS-MxBΔ(1–25) mutants were very poorly expressed, which precludes further examination of their antiviral activity in SupT1 cells. Similar transduction efficiency was observed for MxB, Tat-NLS-MxBΔ(1–25) and Rev-NLS-MxBΔ(1–25), with higher expression of Tat-NLS-MxBΔ(1–25) and Rev-NLS-MxBΔ(1–25) than that of MxB (Figure 6A). When HIV-1(NL4–3) was used to infect SupT1 cells expressing MxB, Tat-NLS-MxBΔ(1–25) or Rev-NLS-MxBΔ(1–25), the number of viral p24-positive cells was markedly reduced, with fold of inhibition of 5.9 for MxB, 3.6 for Tat-NLS-MxBΔ(1–25) and 10.4 for Rev-NLS-MxBΔ(1–25) (Figure 6B). Since HIV-1 develops resistance to MxB inhibition by mutating viral CA protein (Goujon et al., 2013; Kane et al., 2013; Liu et al., 2013; Busnadiego et al., 2014; Miles et al., 2020), we asked whether the MxB-resistant HIV-1 CA mutations also confer resistance to Tat-NLS-MxBΔ(1–25) or Rev-NLS-MxBΔ(1–25). Three MxB-resistant CA mutations were tested, including P90T, E187A and P207S. For the CA mutant P90T, viral infection increased by 68% in MxB-expressing cells, was completely resistant to Tat-NLS-MxBΔ(1–25), and was inhibited by 3.2-folds in Rev-NLS-MxBΔ(1–25) expressing cells (Figure 6B). For the E187A mutant, viral infection was inhibited by 1.6-folds by MxB, increased by 86% in Tat-NLS-MxBΔ(1–25) expressing cells, inhibited by 5.2-folds in Rev-NLS-MxBΔ(1–25) expressing cells (Figure 6B). For the P207S mutant, viral infection was inhibited by 2.7-folds in MxB expressing cells, 1.5-folds in Tat-NLS-MxBΔ(1–25) expressing cells, and 15.7-folds in Rev-NLS-MxBΔ(1–25) expressing cells (Figure 6B). Together, these data showed that Tat-NLS-MxBΔ(1–25) exhibits a restriction profile of HIV-1 CA mutants similar to that of the wild type MxB, whereas Rev-NLS-MxBΔ(1–25) is able to inhibit both the wild type HIV-1 and MxB-resistant CA mutants. It is likely that, due to their arginine-rich nature, the Tat and Rev NLS sequences are able to replace the arginine-rich N-terminal region of MxB without compromising the anti-HIV-1 activity. However, these arginine-rich sequences may differ in their precise interactions with HIV-1 capsid, thus exhibit different sensitivity to viral CA mutations.

## Discussion

In this study, we observed that the bipartite NLS, when being used to replace the N-terminal sequence of MxB, is able to maintain the nuclear envelope localization of MxB. This function is specific for bipartite NLS, since none of the other types of NLS tested in this study is able to restore MxB localization to the nuclear envelope. This unique activity of bipartite NLS may benefit from its ability of directly interacting with importin-β, as opposed to the NLS of SV40 T which needs to interact with importin-α before engaging importin-β, whereas the M9 NLS acts by binding to transportin 1. Alternatively, the bipartite NLS may interact with resident proteins in the nuclear pore complex or on the nuclear envelope so as to anchor MxB to the nuclear envelope.

The bipartite NLS-MxB mutants potently suppress HIV-1 gene expression, at least partly through inhibiting HIV-1 LTR promoter. It is possible that this inhibitory function results from the localization of bipartite NLS-MxB to the nucleus where these MxB mutants either modulate the binding of key transcription factors to HIV-1 LTR promoter DNA or directly interact with specific promoter sequences. Our data showed that this inhibition is specific for HIV-1 LTR, as these bipartite NLS-MxB mutants do not affect the gene expression of the other promoters tested (Figures 4B,D). One possible scenario is that bipartite NLS-MxB has blocked the function of a transcription factor that binds to HIV-1 LTR but not other promoters.

The importance of nuclear envelope localization for MxB function is largely unclear. Residing at the nuclear envelope may be crucial for the cellular functions MxB plays (King et al., 2004), but clearly is dispensable for its anti-HIV-1 activity. This is supported by the results of the Tat-NLS-MxBΔ(1–25) and Rev-NLS-MxBΔ(1–25) which inhibit HIV-1 to a similar degree to that by wild type MxB, yet are mostly dispersed within the cytoplasm and not found on the nuclear envelope. Similarly, the engineered proteins, such as that bear protein dimerization motifs and the N-terminal sequence of MxB, do not reside at the nuclear envelope, but still effectively inhibit HIV-1 (Matreyek et al., 2014; Dicks et al., 2016; Kane et al., 2018).

The N-terminal sequence of MxB possesses the activity of directing protein localization to the nucleus. This is supported by the results showing nuclear location of reporter protein LacZ-GFP when bearing the N-terminal sequence of MxB (Kane et al., 2018). And this activity is dependent on importin-β, since knockdown of importin-β prevents LacZ-GFP/N-MxB from accessing the nucleus (Kane et al., 2018). However, depletion of importin-β did not affect the localization of MxB to the nuclear envelope, suggesting that, although the N-terminal sequence of MxB has the nuclear localization activity, its requirement for MxB localization to the nuclear envelope is independent of importin-β.

Detailed mutagenesis studies showed that the short arginine-rich motif 11-RRR-13 in the N-terminal region is essential for MxB inhibition of HIV-1 (Goujon et al., 2015; Schulte et al., 2015). Coincidentally, the NLS sequences of both Tat and Rev contain arginine clusters, and both exhibit the anti-HIV-1 property when used to replace the N-terminal sequence of MxB. Both structural and biochemical analysis have supported the interaction of MxB N-terminal domain with HIV-1 capsid at the tri-hexamer interface (Smaga et al., 2019; Summers et al., 2019). It is possible that the Tat and Rev NLS enable MxB to bind HIV-1 capsid, but the precise interactions may differ from each other and also differ from that of MxB binding to capsid, since the exact sequences of Tat NLS, Rev NLS and the N-terminal sequence of MxB are different. As a result, one MxB-resistant HIV-1 CA mutation may not necessarily resist the inhibition by Tat-NLS-MxBΔ(1–25) or Rev-NLS-MxBΔ(1–25). Indeed, our data showed that the Rev-NLS-MxBΔ(1–25) strongly inhibits HIV-1 CA mutants that are refractory to wild type MxB and the Tat-NLS-MxBΔ(1–25). This also suggests that Rev-NLS-MxBΔ(1–25) may interact with HIV-1 capsid in a unique fashion which is distinct from either wild type MxB or Tat-NLS-MxBΔ(1–25). Consistent with what observed previously, MxB moderately increased the infection of HIV-1 carrying the P90T mutation in viral capsid (Liu et al., 2013). Interestingly, the Tat-NLS-MxBΔ(1-15) mutant also enhanced the infection of the CA-E187V viral mutant (Figure 6B). It is possible that these capsid mutants have changed the profile of host factors interacting with viral capsid, thus reverse viral response to MxB.

In summary, our results reveal the drastically different effects of various NLS on the subcellular localization and anti-HIV-1 activity of MxB, when used to replace the N-terminal sequence of MxB. Our data further support the essential role of the arginine-rich sequence in targeting HIV-1 capsid structure and thus inhibiting HIV-1 infection, albeit that the exact arginine-rich sequence dictates the specific binding of MxB to viral capsid and hence the resistance profile of HIV-1 capsid.

## Data Availability Statement

The original contributions presented in the study are included in the article/Supplementary Material, further inquiries can be directed to the corresponding author/s.

## Author Contributions

CL and WQ conceived the study and acquired the funding. CL, JT, and KC designed the experiments. KC, ZW, and QP performed the experiments. CL, WQ, KC, ZW, and QP analyzed the data and wrote the manuscript. All authors contributed to the article and approved the submitted version.

## Conflict of Interest

The authors declare that the research was conducted in the absence of any commercial or financial relationships that could be construed as a potential conflict of interest. The reviewer SC declared past co-authorships with the authors to the handling editor.
