# Supplementary material for: Effect of Different Nuclear Localization Signals on the Subcellular Localization and Anti-HIV-1 Function of the MxB Protein
Source: Front Microbiol. 2021 May 20;12:675201. doi: 10.3389/fmicb.2021.675201 (PMC8173038; doi:10.3389/fmicb.2021.675201)
Supplement: Supplementary file 1 [file Table_1.DOCX]

| Species name | Accession number | Species name | Accession number |
| --- | --- | --- | --- |
| Homo sapiens | NP_002454.1 | Bos indicus x Bos taurus | XP_027402441.1 |
| Pan troglodytes | XP_016794045.2 | Bison bison bison | XP_010833243.1 |
| Gorilla gorilla gorilla | XP_004062883.3 | Ovis aries | NP_001072120.1 |
| Pan paniscus | XP_003823954.1 | Canis lupus familiaris | NP_001003133.1 |
| Pongo abelii | XP_002830747.1 | Canis lupus dingo | XP_025317821.1 |
| Nomascus leucogenys | XP_030662121.1 | Myotis brandtii | XP_005885748.1 |
| Macaca mulatta | NP_001073164.1 | Myotis davidii | XP_006754383.1 |
| Macaca fascicularis | XP_005548704.1 | Pteropus alecto | XP_006916792.1 |
| Macaca nemestrina | XP_011724302.1 | Pteropus vampyrus | XP_023393970.1 |
| Cercocebus atys | XP_011892708.1 | Desmodus rotundus | XP_024415590.1 |
| Papio anubis | XP_003895512.2 | Miniopterus natalensis | XP_016076805.1 |
| Theropithecus gelada | XP_025234929.1 | Camelus bactrianus | XP_010956650.1 |
| Chlorocebus sabaeus | XP_007966841.1 | Capra hircus | XP_017907131.1 |
| Rhinopithecus bieti | XP_017735707.1 | Phyllostomus discolor | XP_028360555.1 |
| Rhinopithecus roxellana | XP_010352814.1 | Ursus arctos horribilis | XP_026356450.1 |
| Piliocolobus tephrosceles | XP_023047546.1 | Camelus ferus | XP_006189077.1 |
| Mandrillus leucophaeus | XP_011839576.1 | Galeopterus variegatus | XP_008569440.1 |
| Ailuropoda melanoleuca | XP_011231455.1 | Orycteropus afer afer | XP_007945510.1 |
| Zalophus californianus | XP_027439301.1 | Ceratotherium simum simum | XP_004429766.1 |
| Odobenus rosmarus divergens | XP_004406645.1 | Enhydra lutris kenyoni | XP_022371690.1 |
| Neomonachus schauinslandi | XP_021534845.1 | Loxodonta africana | XP_023414878.1 |
| Callorhinus ursinus | XP_025719219.1 | Propithecus coquereli | XP_012495522.1 |
| Eumetopias jubatus | XP_027982271.1 | Otolemur garnettii | XP_023373873.1 |
| Callithrix jacchus | XP_017822649.1 | Camelus dromedarius | XP_010998684.1 |
| Aotus nancymaae | XP_021525087.1 | Lipotes vexillifer | XP_007452944.1 |
| Cebus capucinus imitator | XP_017394103.1 | Balaenoptera acutorostrata scammoni | XP_007172778.1 |
| Colobus angolensis palliatus | XP_011809447.1 | Equus caballus | XP_005606216.2 |
| Vulpes vulpes | XP_025839146.1 | Equus przewalskii | XP_008536413.1 |
| Saimiri boliviensis boliviensis | XP_010336555.1 | Equus asinus | XP_014694764.1 |
| Tupaia chinensis | XP_006156438.1 | Condylura cristata | XP_012586448.1 |
| Bos taurus | NP_776366.1 | Eptesicus fuscus | XP_028007683.1 |
| Bos mutus | XP_005893078.1 | Bubalus bubalis | XP_006077005.2 |
| Bos indicus | XP_019818391.1 |  |  |

**Supplementary TABLE 1** List of MxB proteins from 65 species
